# Greater temporal changes of sediment microbial community than its waterborne counterpart in Tengchong hot springs, Yunnan Province, China

**DOI:** 10.1038/srep07479

**Published:** 2014-12-19

**Authors:** Shang Wang, Hailiang Dong, Weiguo Hou, Hongchen Jiang, Qiuyuan Huang, Brandon R. Briggs, Liuqin Huang

**Affiliations:** 1 State Key Laboratory of Biogeology and Environmental Geology & Department of Earth Sciences, China University of Geosciences, Beijing 100083, China; 2 Department of Geology and Environmental Earth Science, Miami University, Oxford, OH, 45056, USA; 3 State Key Laboratory of Biogeology and Environmental Geology, China University of Geosciences, Wuhan 430074, China

## Abstract

Temporal variation in geochemistry can cause changes in microbial community structure and diversity. Here we studied temporal changes of microbial communities in Tengchong hot springs of Yunnan Province, China in response to geochemical variations by using microbial and geochemical data collected in January, June and August of 2011. Greater temporal variations were observed in individual taxa than at the whole community structure level. Water and sediment communities exhibited different temporal variation patterns. Water communities were largely stable across three sampling times and dominated by similar microbial lineages: *Hydrogenobaculum* in moderate-temperature acidic springs, *Sulfolobus* in high-temperature acidic springs, and *Hydrogenobacter* in high-temperature circumneutral to alkaline springs. Sediment communities were more diverse and responsive to changing physicochemical conditions. Most of the sediment communities in January and June were similar to those in waters. However, the August sediment community was more diverse and contained more anaerobic heterotrophs than the January and June: *Desulfurella* and *Acidicaldus* in moderate-temperature acidic springs, *Ignisphaera* and *Desulfurococcus* in high-temperature acidic springs, the candidate division OP1 and *Fervidobacterium* in alkaline springs, and *Thermus* and GAL35 in neutral springs. Temporal variations in physicochemical parameters including temperature, pH, and dissolved organic carbon may have triggered the observed microbial community shifts.

Spatial censuses of microbial community structure have been well-studied in diverse hot springs^1^,^2^,^3^,^4^,^5^,^6^,^7^,^8^ and these studies have shown that physicochemical parameters of hot springs such as temperature, pH and nutrient supply are important drivers for shaping microbial diversity and community structure as well as metabolism^4^,^5^,^9^,^10^,^11^,^12^,^13^,^14^,^15^,^16^. In contrast, only a limited number of studies in the last decade have explored temporal changes in hot spring communities^17^,^18^,^19^,^20^,^21^, but similar conclusions have been made that temporal changes in microbial community are correlated with temporal variations of temperature^18^, pH^20^, and nutrient availability^18^,^21^. For example, a previous study showed that a combination of temperature variation and phosphate availability contributed to a temporal difference in microbial diversity in a spring in a tropical geothermal region^18^. Phosphate was the limiting factor for seasonal occurrence of *Chloroflexus* and *Synechococcus*^18^, and these microorganisms were stimulated during the rainy season because of input of an elevated phosphate level in surface runoff into the springs. High rainfall influx in the rainy season was also an important factor for the observed seasonality in the distribution patterns of microbial communities in Tengchong hot springs^19^. In the well-studied Obsidian Pool in the Mud Volcano Area, Yellowstone National Park, the United States, pH variation was correlated to temporal variation in bacterial abundance^20^. Collectively, these past studies have shown that temporal changes in geochemical conditions of hot springs could result in temporal shifts of microbial community structure. Therefore, hot springs that experience temporal variations can provide us with a natural laboratory to study microbial community changes in response to geochemical changes. However, previous studies did not examine any difference in the response pattern of individual microbial lineages to temporal changes in physicochemical conditions.

The hot springs in Tengchong County, Yunnan Province of China, are located in a subtropical area with heavy temporal monsoon rainfall (rainy season May-October), and these springs represent a wide range of microbial niches for highly diverse Achaea, Bacteria, and viruses^9^,^19^,^22^,^23^,^24^,^25^,^26^. Rehai and Diantan (formally Ruidian) are two main geothermal areas within the Tengchong geothermal system. A previous study showed that Rehai springs contained similar sediment and water communities in winter, while Diantan sediment communities harbored very different communities from those in water^9^. Despite these results, little is known about how microbial community structure changes over time in these springs, and if this change is similar between water and sediment. The effects of environmental change on microbial community structure are, to some extent, dependent on the community of interest; in other words, a subset of a community and the entire community structure may respond to different environmental parameters. For example, a previous study showed that significant seasonal patterns were observed for some individual species but not for the entire community structure^18^. In the Tengchong hot springs, dominant species were similar between sediment and water in winter season^19^, but no systematic studies have been performed to compare water and sediment communities in response to temporal geochemical changes. Hence, it is imperative to compare and contrast temporal variations in water and sediment microbial diversity and community structure in Tengchong hot springs, at different taxonomic levels (whole community and individual microbial lineages).

Therefore, temporal changes of microbial communities across three sampling time points in the Tengchong hot springs were studied by using high-throughput sequencing of 16S rRNA genes integrated with extensive geochemical analyses. The goals of this study were to 1) determine the temporal variability of microbial lineages in individual hot springs in correlation with geochemical conditions; and 2) compare the response patterns between sediment and water communities to temporal variations in geochemistry.

## Methods

### Sampling sites description

Three field trips were made to Tengchong, Yunnan Province, China in 2011: one in the dry season (January) and two in the rainy season (June and August). A total of nine springs across a range of pH and temperature were chosen (see map in Hou et al. 2013^9^ or Briggs et al. 2013^19^), including 7 from Rehai and 2 from Diantan (formally called Ruidian) to collect a total of 49 samples (28 sediments and 21 water samples). Rehai and Diantan geothermal fields are about 60 kilometers away from each other. Large pools with circumneutral pH such as Gongxiaoshe (Gxs) and Jinze (Jz) are located in the Diantan geothermal field. Rehai harbors various types of hot springs: small source, high discharge springs such as Gumingquan (Gmq) and Jiemeiquan (Jmq); small, shallow acidic mud pools, such as those in Diretiyanqu (Drty-1, Drty-2 and Drty-3) that formed a decreasing temperature gradient; shallow acidic pool Zhenzhuquan (Zzq); and shallow spring with multiple geothermal sources such as Shuirebaozha (Srbz). Not all the sediment and water samples were paired. In Diretiyanqu area, we could not collect any water samples in June and August because waters and sediments were well-mixed in the shallow mud pools due to heavy rainfall. Therefore, there were no water samples for Drty-2 in June and August and for Drty-3 in August.

### Field measurements and sample collection

Concentrations of ammonium (NH_4_^+^), total sulfide (ΣS^2−^), ferrous iron (Fe^2+^), nitrate (NO_3_^−^), and nitrite (NO_2_^−^) were measured in the field with spectrophotometric Hach kits (Hach Chemical Co., IA, USA). Water samples for laboratory measurements, e.g., cations, anions, dissolved organic carbon (DOC), and total nitrogen (TN), were collected by filtration of spring water through 0.45 μm Whatman GF/F filter. Water samples for DOC and cations were acidified and stored at 4°C. Depending on spring turbidity, various volumes of water were filtered through the 0.22 μm syringe polyethersulfone (PES) membrane filters (Pall Corp., NY) to collect biomass.

Sediment samples were collected with sterile spatulas and spoons, and homogenized in a pre-sterilized aluminum pan and then placed into 50 mL sterilized Teflon tubes. Sediment samples for geochemical analyses, including total organic carbon (TOC) and mineralogy were stored on ice until analyses^9^,^19^. All samples for microbiological analyses (sediments and biomass-containing filters) were immediately frozen in liquid nitrogen or dry ice, stored on dry ice during transportation, and at −80°C in the laboratory until analysis.

### Laboratory geochemical analyses

Geochemical data for the January and June samples have been reported in other publications^9^,^19^. For the August samples, the same analytical procedures were used for water and sediment geochemical analyses. Cation and anion concentrations were measured by using ion chromatography (Dionex DX-600, USA), and DOC and TN concentrations were measured on a multi N/C 2100S analyzer (Analytik Jena, Germany). Sediment samples for TOC and mineralogy measurements were dried and ground. TOC samples were treated with 1 N HCl overnight to remove carbonates and then washed to a neutral pH followed by analysis with the multi N/C 2100S analyzer. Samples for quantitative mineralogy were X-ray scanned from 2 to 70 degree two theta with Cu K-alpha radiation (40 kV, 35 mA).

### DNA extraction and pyrosequencing

DNA was extracted from biomass-containing filters or from 0.5 g sediment samples using the FastDNA SPIN Kit for Soil (MP Biomedical, OH, USA). The extracted DNA was amplified using a universal modified primer set 515F (5′-GTGYCAGCMGCCGCGGTAA-3′)-1391R (5′-GACGGGCGGTGWGTRCA-3′) as previously described^9^. Unique 8-bp barcodes for each sample were added at the 5′-end of both the forward and reverse primers to demultiplex sequences. Each 25 ul PCR system contained 10 ng template DNA, 1×PCR buffer, 400 nM each primer, 200 μM dNTPs, and 0.3 unit rTaq polymerase (Takara, Dalian, China). PCR and purification of the amplified products were performed as previously described^9^. Pyrosequencing was performed with a 454 GS FLX Titanium technology (454 Life Sciences, Branford, CT, USA) at the Chinese National Human Genome Center in Shanghai.

### Data processing and statistic analyses

The sequence data for the January samples have been used in another publication^9^ and data for the June and August samples were original. All these data were processed together with new methods. Sequence demultiplexing and quality control were performed in Mothur^27^ and QIIME^28^. Sequences that had an average quality score of lower than 27 over a window size of 50 nt were removed from subsequent analysis. OTU clusters at 97% sequence identity were determined by the using UCLUST algorithm^29^. The first sequence from each OTU was picked as a representative and taxonomy was assigned to each representative using the ribosome database project (RDP) classifier algorithm^30^. Sequences that could not be classified with this algorithm were manually searched against the NCBI BLAST database using BLASTN^31^ to find highly similar hits. Following a recent recommendation^32^ we treated GAL35 as a distinct lineage, rather than a class in the candidate phylum OP1. Alpha diversity (within samples) and beta diversity (among samples) were calculated using species-level operational taxonomic units (OTUs) (at the 97% identity level) in QIIME as previously described^9^. Data used for alpha diversity calculations were normalized by randomly sub-sampling 1000 sequences in each sample with 1000 replicates to minimize the effects caused by different sequencing efforts. Any samples with <1000 sequences were excluded from this analysis. A variety of alpha diversity indices were calculated including Chao1 (a measure of richness, namely the estimated number of phylotypes), Shannon (includes both richness and evenness), Equitability (i.e. evenness, distribution of phylotypes), and phylogenetic diversity^33^ (PD-phylogenetic closeness in a subset of phylotypes).

The non-metric dimensional scaling (NMDS) ordination with 500 random starts and clustering tree were built to depict the community structure based on the Bray-Curtis dissimilarity matrix of detected OTUs in the R package ‘Vegan'. Analyses of similarity (ANOSIM), non-parametric multivariate ANOVA (ADONIS), and multi-response permutation procedure (MRPP) were performed to test for significant differences in microbial community composition between different sampling time points (i.e., January vs. June vs. August), sample types (i.e., sediment vs. water), and pH ranges (i.e., acidic vs. alkaline vs. neutral). SIMPER (similarity percentage) analysis was performed to rank the taxa that contributed to the differences among the various groups described above. The average abundances of those top ranked taxa in each group were then calculated. The Envfit function was used to overlay the most significant environmental variables on the NMDS ordination. The pyrosequencing reads were deposited to the Short Read Archive database at NCBI (for January data: Accession No. SRA060322; for June and August data: Accession No. SRA177186).

## Results

### Water and sediment geochemistry

Consistent with previous results^19^, DOC and TN of spring waters and TOC of sediments were higher in samples of the rainy season (June and August) than in those from the dry season (January), reflecting the increased monthly precipitation from January to August (Table 1). Acidic springs and neutral-alkaline springs exhibited different water chemistry. In all three sampling times moderate-temperature acidic springs were dominated by high levels of sulfate and ferrous ion followed by calcium and potassium (Table S1 and Fig. S1a). The concentrations of these ions increased from January to June, and then decreased in August. In neutral-alkaline springs, waters exhibited high levels of chloride and sodium concentrations followed by less abundant potassium and calcium (Table S1 and Fig. S1b). Chloride concentration decreased from January to June to August, whereas the concentrations of cations (potassium, sodium and calcium) generally increased from January to June, but decreased in August samples (Table S1 and Fig. S1b).

There were no uniform temporal trends of changes among ions in high-temperature acidic springs (Table S1 and Fig. S1c). These spring water experienced great temporal variations in physicochemical conditions (Table 1, Table S1 and Fig. S1c). For example, pH in Zhenzhuquan (Zzq) ranged from 4.8 in January to 4.7 in June to 6.1 in August (Table 1). In this spring, Fe^2+^ and SO_4_^2−^ concentrations decreased from January to August (Table S1 and Fig.S1c), possibly caused by rainwater dilution and increase of pH. However, in Diretiyanqu-1 (Drty-1) where pH also increased from January to August, Fe^2+^ increased in June and then decreased in August (Fig. S1c), indicating other Fe^2+^ supply sources in June, such as surface runoff and microbial activity (i.e. ferric iron reduction). Notably, potassium in Diretiyanqu-1 (Drty-1) from August was much higher than any other springs at any of the sampling times (Table S1 and Fig. S1c).

The different springs also experienced temporal changes in nitrogen species (Table S1 and Fig. S2). Acidic springs and most of the neutral-alkaline springs (except Shuirebaozha (SrbzD) and Gongxiaoshe (Gxs) springs) had higher concentrations of NH_4_^+^ in the rainy season (Fig. S2). In Shuirebaozha (SrbzD), NH_4_^+^ progressively decreased from 10.7 μM in January to < 1 μM in August. In Gongxiaoshe (GxsB) NH_4_^+^ decreased in June and then slightly increased in August. In contrast to NH_4_^+^, NO_2_^−^+NO_3_^−^ concentrations in most of the springs (except Gumingquan (Gmq) and Zhenzhuquan (Zzq)) were higher in January than in June and August. However, in Gumingquan (GmqP), NO_2_^−^+NO_3_^−^ concentration increased in June and dropped to <1 μM in August. The NO_2_^−^+NO_3_^−^ concentration in Zhenzhuquan (Zzq) remained <0.5 μM in all three sampling times.

Three groups of major minerals in hot spring sediments were identified (quartz, K-feldspar, and clay minerals such as kaolinite, smectite and illite) (Table 2); however, abundance of these minerals differed between acidic and circumneutral-alkaline springs: Quartz and kaolinite were more abundant in acidic springs, whereas more K-feldspar was detected in circumneutral-alkaline springs. Clay minerals were comparable in all the investigated springs except Gongxiaoshe spring (GxsB), where aragonite was the predominant mineral (Table 2).

### Microbial diversity

**Figure 1:**
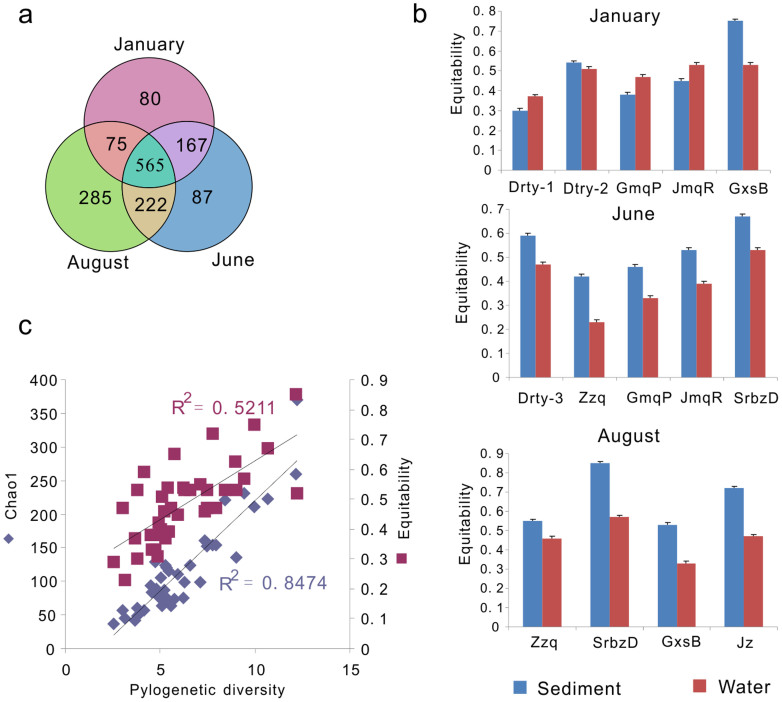
Temporal changes in microbial diversity. (a). Microbial community relatedness between different sampling time points. More taxa were observed in the rainy season June and August, and the August community also harbored the largest number of unique taxa (n = 285, only occurred at one sampling time point). (b). Phylogenetic diversity in paired sediment and water samples for January, June and August. (c). Positively linear relationship between richness (i.e. Chao1) and phylogenetic diversity, as well as between evenness (i.e. Equitability) and phylogenetic diversity.

Both hot spring sediment and water experienced large temporal variations in microbial diversity. All phylotypes collected from all sites were grouped according to sampling time to evaluate temporal effects on microbial diversity (Fig. 1 and Table S2). We found that more phylotypes were observed in the rainy season, especially in August (Fig. 1a). Furthermore, the August community (n = 285) contained more unique taxa (the OTUs only found in one sampling time point) than January (n = 80) and June (n = 87) (Fig. 1a). The temporal effect could also be observed in equitability, with higher equitability values in sediment than its water counterpart in the rainy season (June and August), but in the dry season (January) the equitability values were similar between sediment and water (except in GxsB) (Fig. 1b). Positively linear relationship between richness (Chao1) and phylogenetic diversity, as well as between evenness (Equitability) and phylogenetic diversity were observed (Fig. 1c), which suggested sediment community in the rainy season, especially in August, was composed of a group of highly diverse microbes.

### Limited temporal changes observed for the entire microbial community structure

**Figure 2:**
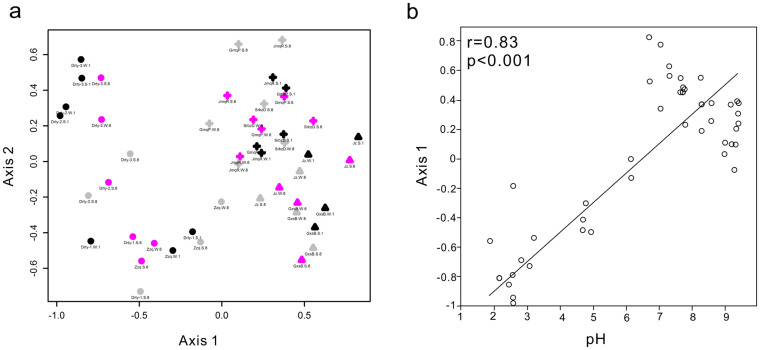
pH-dependent microbial community distribution pattern. (a). NMDS ordination for all the samples collected during three sampling time points. Different colors represent three sampling time points, black for January, pink for June and grey for August. Different symbols indicate samples from different pH range, circles for acid samples, crosses for alkaline samples, and triangles for neutral samples. This figure shows a pH-dependent clustering pattern of spring samples, but not according to sampling time. (b). Strong linear correlation between NMDS axis 1 and pH.

Different statistical analyses were employed to evaluate the temporal effects on the entire microbial community structure, but the results were dependent on the methods used. Although more phylotypes were observed in the rainy season, their abundances were so low (Figure S3, the “rare lineages” category) that their emergence could not be detected by the statistical methods (MRPP, ANODIS, and ANOSIM). Therefore the statistical results barely showed significant change in the entire community structure across the three sampling times (Table 3, Jan vs. Jun vs. Aug, p>0.05). The communities were predominantly composed of phyla *Crenarchaeota* (30%~35% of the total sequences in each group) and *Aquificae* (43%~48%) across January, June and August (Fig. S3). However, temporal changes in some springs were observed when NMDS ordinations were used to visualize the temporal difference in microbial community structure (Figs. 2a & S4). In this case, pH was an important factor in shaping the microbial community distribution patterns (Fig. 2b). The pH effect was also confirmed by the significance tests (Table 3, acidic vs. neutral vs. alkaline, p<0.05).

When we further investigated the microbial community structure separately for each sampling time, limited changes were observed from January and June to August (Fig. S4). For example, acidic springs formed two distinct clusters according to temperature in both January and June (Fig. S4a and S4b) but in August no temperature-dependent clusters were observed for acidic springs. Furthermore two high-temperature acidic springs (Zhenzhuquan and Diretiyanqu-1) were far apart from each other in August (Fig. S4c). Some other environmental parameters that constrained the microbial distribution patterns were identified, such as total organic carbon in sediments and nitrogen species in water (Fig. S4). Major chemical ions such as sulfate, ferrous iron, sodium, chloride and fluoride also showed significant correlations with microbial community distribution patterns; however, they were also co-varied with pH, making it difficult to distinguish between pH and these chemical ion effects.

### Greater temporal changes in sediment community than water community

**Figure 3:**
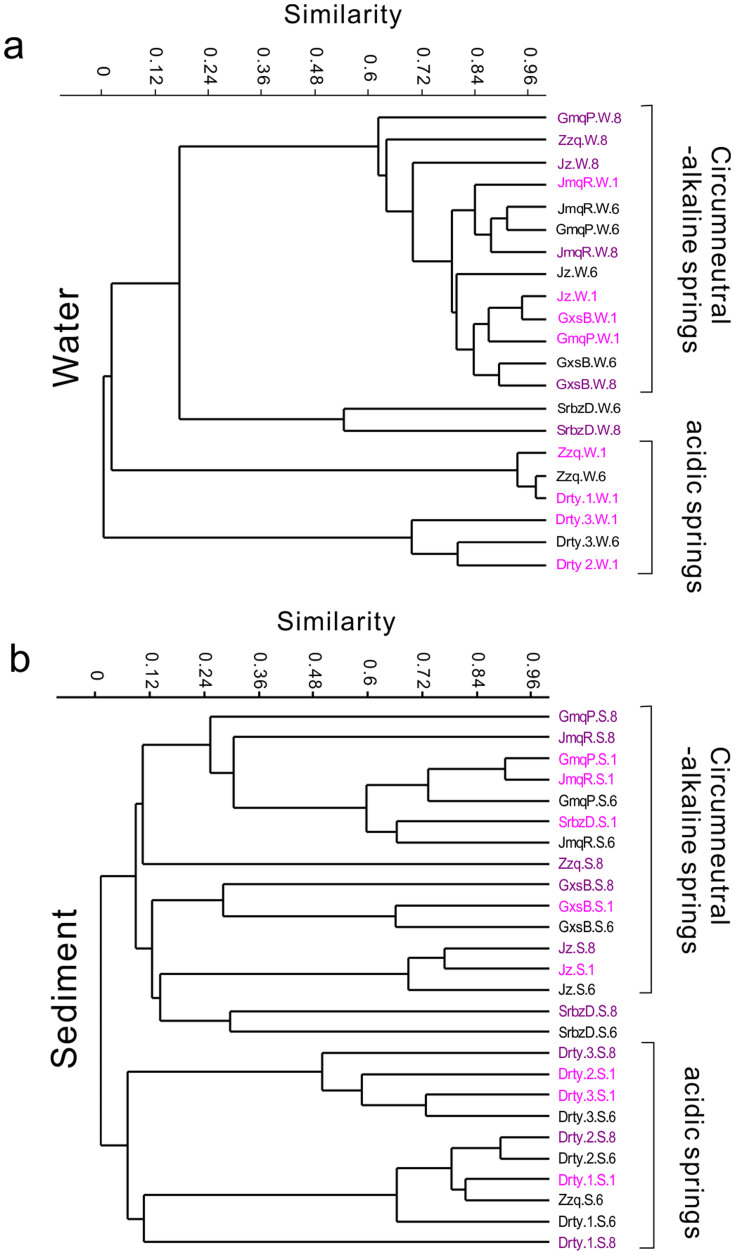
Clustering patterns for the sediments and water samples, respectively. (a). A cluster tree for water communities (denoted by “W” after the abbreviation for spring name). (b). A cluster tree for sediment communities (denoted by “S” after the abbreviation for spring name). All sample names are coded in three different colors, corresponding to three different sampling time points (e.g., black for January, pink for June and purple for August, and also post-fixed with 1, 6, and 8).

**Figure 4:**
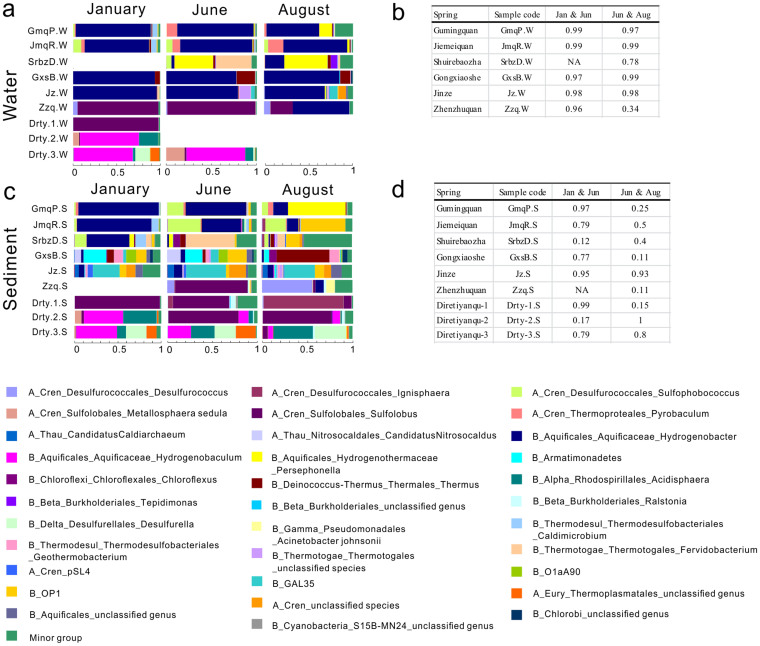
Temporal changes in microbial community structure in individual springs. (a) and (c). Taxonomic compositions in individual springs at the genus level for water and sediment samples, respectively. “Minor group” represents the sum of microbial lineages with relative abundance <5%. (b) and (d). Correlations between January and June microbial community structures, as well as between June and August microbial community structures as revealed by Mantel test with all p-values <0.01. Higher correlation values indicated more similar microbial community structures between two sampling times.

Although limited temporal changes were observed in the entire community across the three sampling times, great temporal variations were observed when the whole community was separated into water and sediment communities (Fig. 3, Fig 4 and Table 4). The overall average dissimilarity for the sediment communities across January, June and August was 83.54%, much higher than the overall average dissimilarity of 50.21% for the water communities over the same time period (Table 4). This difference suggested that sediment community displayed more pronounced temporal change than water community. A similar pattern was also revealed on the clustering tree: short branches indicated a high similarity (the lowest Bray-Curtis similarity value was >0.6, except Shuirebaozha spring) between the water communities from the three sampling times (Fig. 3a), whereas a low similarity (the lowest value was about 0.1) was observed between the three sediment communities (Fig. 3b). SIMPER analysis was performed to rank the contribution of individual taxa to the observed temporal differences in microbial communities (Table 4). The results showed that the lower dissimilarity for the water communities across January, June and August could be ascribed to relatively small abundance changes of top 14 taxa (contribution >1%) across the three sampling times, whereas the higher dissimilarity for the sediment communities was due to larger abundance changes of top 22 taxa (contribution >1%) across the same time period (Table 4).

When examined for individual springs, the dominant taxa within the water community did not show pronounced temporal changes (Fig. 3a, Fig. 4a and 4b) and the Mantel test confirmed the significant similarities (p<0.01) in water communities across different sampling times (Fig. 4b). The circumneutral-alkaline spring community was mainly composed of *Hydrogenobacter* (59%~96% of total sequences in each spring) (Fig. 4a), except the Shuirebaozha spring (SrbzD.W). The dominant taxa in the Shuirebaozha spring changed from *Persephonella* and *Fervidobacterium* in June to *Persephonella* and *Hydrogenobacter* in August (Fig. 4a). In acidic springs, Zhenzhuquan (Zzq.W) was the only spring from which water samples were collected at all three time points. In this spring, communities between January and June were highly similar to each other (Fig. 4b) and both were predominated by *Sulfolobus* (Fig. 4a), but the August communities were significantly different from January and June (Fig. 4b), with *Hydrogenobacter* being the most abundant taxon in August followed by *Sulfolobus* (Fig. 4a).

In sediments, specific microbial lineages exhibited major changes across the three sampling times, especially from June to August (Fig. 3b, Fig. 4c and 4d), except the Jinze spring (Jz.S), where the sediment community composition remained similar across the three sampling times. In the two alkaline springs Gumingquan (GmqP.S) and Jiemeiquan (JmqR.S) which were characterized by fast-flowing and high discharges, the Mantel test showed high similarity (Mantel r = 0.97 for Gumingquan and 0.79 for Jiemeiquan) between January and June sediment community structures (Fig. 4d). A single lineage *Hydrogenobacter* was the dominant constituent in both January and June communities but *Sulfophobococcus* emerged in June (Fig. 4c). Low similarity (Mantel r = 0.25 for Gumingquan and 0.5 for Jiemeiquan) was observed between June and August community structures (Fig. 4d), largely because *Persephonella* and OP1 became the dominant member in August Gumingquan and Jiemeiquan sediments, respectively (Fig. 4c). Likewise, the alkaline Shuirebaozha spring also displayed a strong temporal variation in its sediment microbial community structure (Fig. 4c and 4d).

Similar to these alkaline springs, high-temperature acidic springs Zhenzhuquan (Zzq.S) and Diretiyan-1 (Drty-1.S) also exhibited important temporal changes in sediment community structures. *Sulfolobus* was the dominant member in these two springs in both January and June communities, but in the August community, *Desulfococcus* substituted *Sulfolobus* in Zhenzhuquan and *Ignispaera* replaced *Sulfolobus* in Diretiyan-1 (Fig. 4c). This change was confirmed by the Mantel test showing a high similarity between January and June but a low similarity between June and August community structures (Fig. 4d). However, a slight temporal change in moderate-temperature acidic spring Diretiyanqu-3 (Drty-3.S) community structure was observed. In Diretiyanqu-3, the abundance of *Hydrogenobaculum* decreased over time (47% in January vs. 26% in June vs. 5% in August), but those of *Acidicaldus* (10% vs. 26% vs. 43%) and *Desulfurella* (24% vs. 24% vs. 34%) increased (Fig. 4c). Another moderate-temperature acidic spring, Diretiyanqu-2 (Drty-2.S), was an exception to the above observations, e.g., the June and August community structures were similar to each other, but they were different from that in January (Fig. 4c and 4d).

Similar to a previous microbial census study in January^9^, Diantan sediments contained more diverse microbial lineages than Rehai sediments. Dramatic temporal changes were observed for the Gongxiaoshe spring sediments (GxsB.S), especially in August (Fig. 4c and 4d). Specifically, the neutral Gongxiaoshe spring contained similar microbial lineages in January and June, including Candidatus *Nitrosocaldus*, *Armatimonadetes*, *Thermus*, *Geothermobacterium*, GAL35, O1aA90 and OP1 (Fig. 4c). However, in August, a single dominant lineage *Thermus* emerged, followed by less abundant *Geothermonbacterium* and *Chloroflexus*, and this large change in microbial community structure from June to August was confirmed by the extremely low value of 0.11 in the Mantel test (Fig. 4d).

## Discussion

Previous studies on Tengchong hot springs using pyrosequencing^9^ and Phylochip^19^ have provided a census of overall microbial community and temporal change (January vs. June). According to those results, non-endemic microbes which were flushed into springs due to enhanced rain influx may have accounted for the temporal changes in microbial community distribution pattern from January to June^19^. With the use of the pyrosequencing method and one more sampling time point (January vs. June vs. August), we were not only able to further document the temporal variability of the overall hot spring communities, but also to reveal the temporal changes of individual genera in individual springs, and compare the temporal variability between sediments and water communities. The exact reasons accounting for the temporal changes in microbial community structure were difficult to establish, but the results from temporal changes of geochemistry in combination with the physiology of the culture representatives could shed light on these observed temporal changes of individual lineages.

Our data indicated that pH was a primary factor in determining the overall community assembly across the studied time points, followed by temperature and DOC. Temporal changes in these geochemical parameters may have induced microbial community shifts. For example, the dominant microbial lineages in the high-temperature acidic Zhenzhuquan spring (Zzq) changed from *Sulfolobus* in both sediments and water in January and June to microaerophilic *Hydrogenobacter* in the water and anaerobic *Desulfococcus* in the sediments in August. This significant change was possibly due to the pH increase from 4.7~4.8 in January and June to 6.1 in August. This high pH in August exceeded the growth pH range for *Sulfolobus* (0.9~5.8)^34^, but fit well with the pH range of *Hydrogenobacter* (near neutral pH)^35^ and *Desulfococcus* (pH 6.0~6.5)^36^.

The reasons for the decrease of *Sulfolobus* in another high-temperature acidic spring, Diretiyanqu-1, may be different. In this spring, pH also increased over time (from 2.6 to 5.0), but did not exceed the upper pH limit for *Sulfolobus*. Instead, high ion accumulations of NH_4_^+^, K^+^, and Na^+^ in August could have inhibited the growth of *Sulfolobus* species by affecting its RNA polymerase activity^37^,^38^,^39^. Indeed, NH_4_^+^ (1.3 mM) and K^+^ (9.4 mM) concentrations in August were much higher than those in natural environments where *Sulfolobus* *solfataricus* have been isolated (1 mM for both ions)^39^. In addition, the high concentration of DOC in Diretiyanqu-1 observed in August would have facilitated the rapid growth of chemoorganotrophic microorganisms such as *Ignisphaera*. In these two high-temperature acidic springs (Zzq and Drty-1), due to these temporal changes of microbial lineages, microbial functions, as inferred from the microbial physiology, could have changed correspondingly: dominance of sulfur-oxidation in January and June by *Sulfolobus*-related species^34^ to sulfur/sulfate-reduction by *Desulfococcus*^40^ and *Ignisphaera*^41^ related species in August. This functional shift was supported by the observed decrease in sulfate concentration from January to June to August.

Consistent with our previous results^9^, our data suggest that several genera of *Desulfococcales* tolerated a wider range of pH than their optimal pH conditions. For example, a previous study showed that *Ignisphaera* is a moderate acidophile with a pH range of 5.4 to 7.0 (optimum pH 6.4)^41^. However, this genus was present in acidic Tengchong springs with pH <5. Likewise, culture-independent studies detected 16S rRNA sequences related to *Ignisphaera* in a Great Basin spring with pH 7.2^42^ although its abundance was low. Another lineage *Sulfophobococcus* was previously reported in environments with a pH range of 6.5~8.5 and a low ion strength (<0.2% w/v NaCl)^43^, however, this lineage was detected in alkaline Tengchong springs with pH ≥ 9. Similarly, *Desulfurella*, which was considered as a neutrophile^44^, was found in acidic springs in the present study, co-existing with *Hydrogenobaculum* and such co-occurrence has been observed in another acidic spring, i.e. Dragon spring in Yellowstone National Park (YNP)^45^. Collectively, these results suggest that some uncultured members within these lineages can tolerate a broader pH range in complex natural environments than in defined culture media^38^,^43^. Furthermore, microbes in natural environments can be present in micro-environments within sediments or surrounded by their own cell aggregates to avoid unfavorable conditions. For example, *Sulfophoboccus*^43^ and *Ignisphaera*^41^ were reported to create aggregates, so this mechanism may help them inhabit higher and lower pH environments, respectively, than their optimal pH conditions.

In addition to pH and water chemistry, a large temperature change also could induce microbial change. For example, in Gumingquan, the sediment community shifted from *Hydrogenobacter* in January and June to *Persephonella* in August. This shift corresponded to a temperature decrease (from 93°C in January to 83°C in June, to 69°C in August). The dominance of *Persephonella* at a moderate temperature e.g. 69°C was consistent with the recent results showing that *Persephonella* was isolated from worldwide springs with temperature no more than 70°C^46^. Members in *Persephonella*^47^ and *Hydrogenobacter*^48^ are all obligate H_2_-oxidizing chemolithotrophs, suggesting that chemolithotrophic growth was a dominant process in Gumingquan for all three sampling times. Overall, these results confirmed that time-course changes of microbial species were associated with the changing environmental conditions^49^. These results are consistent with previous studies in hot springs that microbial community structure and diversity in hot springs were significantly affected by physicochemical parameters such as pH, temperature and water/sediments chemistry^3^,^4^,^9^,^10^,^19^,^35^,^50^,^51^,^52^,^53^,^54^.

It is unexpected that sediment communities exhibited more dramatic temporal changes than their water counterparts. To our knowledge, only one other study in a Great Basin spring of the United States showed greater temporal change in sediment community than in water^10^. The major change observed in the sediment communities of Tengchong was the flourish of heterotrophs or fermenters in August sediments, suggesting that the elevated bioavailable nutrients in the August sediments may have favored the growth of heterotrophs. A previous study has shown that nutrient amendment can drive dramatic microbial community shifts in a groundwater ecosystem^55^, and our results suggest that similar shifts are possible in hot springs. Indeed, DOC in June and August were much higher than that in January, apparently due to high rainfall in June and August. However, it is interesting to note that despite high DOC in June, the sediment community structures did not shift until August. This lag in their response time suggests that sediment microbial communities need time to acclimate themselves to the new geochemical conditions. This kind of lag time is commonly observed when microorganisms collected from natural environments are cultivated in laboratory defined media. The length of lag time is dependent on how different the new condition is from the original one. A previous cultivation study of thermophilic *Nitrospira* reported that the lag time ranged from 2 to 4 months depending on different NO_2_^−^ concentrations used in media^56^. In comparison, our results suggested a fairly rapid adaption of sediment microbial community with a time-lag period of only a few months (May to August, May is the start of rainy season, but no samples were collected). However, our insufficient sampling time resolution did not allow any quantitative estimate of this lag time. More frequent sampling will be conducted to confirm this preliminary finding.

Although heavy surface runoff in the rainy season could have brought soil microbes into hot springs, our results showed that the abundant heterotrophs in the August sediments were all hyperthermophiles or thermophiles, which were not likely to be derived from surrounding soil environments. Our results are consistent with a recent lipid biomarker study where glycerol dialkyl glycerol tetraether from spring sediments were distinctly different from surrounding soils (Wu et al., personal communication). These heterotrophs occurring in August grew either by anaerobic respiration (such as *Desulfococcus*^36^, *Acidicladus*^57^ and *Desulfurella*^44^ using sulfate, ferric iron and sulfur as an electron acceptor, respectively) or fermentation (such as *Ignisphaera*^41^, *Thermus*^58^, *Fervidobacterium*^15^, *Sulfophobococcus*^43^ and GAL35^32^). These physiological characteristics suggest that they may have initially inhabited heterogeneous and anaerobic micro-environments within spring sediments in January, but when the conditions became favorable (e.g., high TOC and nutrients), these minor microbes may have flourished and even become predominant. If so, this speculation would support a hypothesis that is widely applied to marine environments: “rare biosphere” acts as a seed bank in terms of the whole community or their genes^59^,^60^.

Relative to sediment communities, water communities did not show much temporal change. We speculate that this was likely due to fundamental differences between sediment and water environment. In hot spring sediments micro-niches and physical barriers can host diverse and heterogeneous microbial communities which would result in high richness and evenness. High richness, evenness, and phylogenetic diversity in sediment, especially in the rainy season, may suggest a more complex and dynamic sediment community that interacts with the changing environmental conditions. Changes in geochemical conditions may cause previously dominant members to diminish and previously minor members to flourish, because minor members under previously sub-optimal conditions may find new conditions to be optimal for their growth. However, in water, constant mixing and lack of physical barrier would result in less diverse microbial community. Future work is necessary to focus on temporal changes at the functional/activity level with a higher temporal resolution.

## Conclusions

Limited temporal changes were observed in the entire community across springs and sampling times, but greater temporal changes were observed for specific microbial lineages in individual springs, likely because of the higher sensitivity of these genera to environmental changes. Water and sediment communities responded differently to temporal physicochemical changes. Whereas water communities were stable from January to June to August, sediment communities were more responsive to temporal geochemical changes. Specifically more abundant anaerobic heterotrophs and/or fermenters occurred in August sediment relative to January and June. The hyperthermophilic and thermophilic nature of these heterotrophs suggests that they were not transported into hot springs from the surroundings by increased surface runoff in August, but rather their occurrence or even dominance was due to large temporal variations of physicochemical conditions (i.e. pH, temperature and DOC) in these springs.

## Supplementary Material

Supplementary Information Supplementary tables and figures

## Figures and Tables

**Table 1:** Physicochemical conditions of Tengchong hot springs

Spring name	Spring ID^a^,b	pH	Temperature	DOC_W	TN_W (mg/L)	TOC_S	SampleType
			(°C)	(mg/L)		(%)	
Diretiyan-1	Drty-1.1	2.6	85.1	43.9	16.8	0.1	Sed&Water
Diretiyan-2	Drty-2.1	2.6	64.5	11.1	2.8	0.2	Sed&Water
Diretiyan-3	Drty-3.1	2.5	55.1	9.8	4.5	0.3	Sed&Water
Gumingquan_Pool	GmqP.1	9.4	93	1.7	0.4	0.1	Sed&Water
Jiemeiquan_Right	JmqR.1	9.4	83.2	1.7	0.3	0.1	Sed&Water
Zhenzhuquan	Zzq.1	4.8	89.1	1.9	4.1	0.1	Water
Shuirebaozha_Downstrem	SrbzD.1	8.3	78.2	1.7	0.4	0	Sed
Gongxiaoshe_Bottom	GxsB.1	7.3	73.8	1.5	0.4	2.8	Sed&Water
Jinze	Jz.1	6.7	81.6	1.6	0.4	2.5	Sed&Water
Diretiyan-1	Drty-1.6	3.2	87.8	117.6	28.8	0.4 ± 0.04	Sed
Diretiyan-2	Drty-2.6	2.8	66.3	7.4	3.8	1.6 ± 0.06	Sed
Diretiyan-3	Drty-3.6	3.1	53	7.4 ± 0.02	3.8	NA	Sed&Water
Gumingquan_Pool	GmqP.6	9.4	83.5	NA	NA	0.4 ± 0.03	Sed&Water
Jiemeiquan_Right	JmqR.6	9	84.7	10.6	0.3 ± 0.07	0.6 ± 0.02	Sed&Water
Zhenzhuquan	Zzq.6	4.7	92.1	NA	NA	0.6 ± 0.06	Sed&Water
Shuirebaozha_Downstrem	SrbzD.6	8.3	72.1	1.6	0.3	1.1 ± 0.08	Sed&Water
Gongxiaoshe_Bottom	GxsB.6	7.7	75	8.3	0.4	3.69 ± 0.13	Sed&Water
Jinze	Jz.6	7	80.7	6.5	0.2 ± 0.06	4.5 ± 0.11	Sed&Water
Diretiyan-1	Drty-1.8	4.9	87.6	127.5 ± 0.15	39.9 ± 1.74	NA	Sed
Diretiyan-2	Drty-2.8	2.2	68.5	21.8 ± 0.11	18.6 ± 0.18	0.6 ± 0.15	Sed
Diretiyan-3	Drty-3.8	1.9	56.5	15.4 ± 1.76	18.1 ± 0.01	NA	Sed
Gumingquan_Pool	GmqP.8	9.3	69	12.8 ± 0.71	1.8 ± 0.06	0.4 ± 0.02	Sed&Water
Jiemeiquan_Right	JmqR.8	9.2	82.1	7.4 ± 0.16	1.7 ± 0.01	0.4 ± 0.01	Sed&Water
Zhenzhuquan	Zzq.8	6.1	91	12.5 ± 1.19	1.8 ± 0.16	0.5 ± 0.03	Sed&Water
Shuirebaozha_Downstrem	SrbzD.8	8.6	66.6	10.7 ± 0.25	2 ± 0.05	0.6 ± 0.05	Sed&Water
Gongxiaoshe_Bottom	GxsB.8	7.6	76.5	NA	NA	3.9 ± 0.08	Sed&Water
Jinze	Jz.8	7.8	82.4	NA	NA	1.2 ± 0.04	Sed&Water

^a^Sample IDs are composed of abbreviations of spring name and sampling month. “.1” denotes January, “.6” June and “.8” August. e.g., Drty-1.1 denotes sample collected from Diretiyanqu-1 spring in January 2011.

^b^Monthly averaged solar irradiation and precipitation for January, June and August were 438.86, 438.87 and 433.08 MJ/m^2^ and 69.9, 157.3 and 197.5 mm, respectively. Sampling trips were during January 6~ January 10, June 6 ~ June 10, and August 5 ~ August 9, 2011.

**Table 2:** Quantitative mineralogy in June sediments as determined by X-ray diffraction (unit: %)

SampleID	Quartz	K-feldspar	Albite	Calcite	Aragonite	Dolomite	Halite
Drty-1.6	52.7	3.8	2.9	1.4		1.4	0.8
Drty-2.6	44.7	9.7	4	1.7		1.7	0.6
Drty-3.6	44.9	5.7	5.1	0.6			0.4
GmqP.6	23.8	38.7	4	2.5		0.8	0.6
JmqR.6	14.6	39.9	2.1	3.4		1.1	1.3
Zzq.6	9.4	46.2	0.7		2.9	1.1	0.9
SrbzD.6	27.8	26.9	4.4				0.2
GxsB.6			1.5	12.1	86.3		
Jz.6	20.7	19.7	3.7	12.3	0.6	0.7	0.3

SampleID	Gypsum	Barite	Sulfur	Kaolinite	Smectite	Illite	Biotite
Drty-1.6	2.4	1	0.6	9.5	8.4	9.6	5.5
Drty-2.6	1.6	0.8	0.9	14.9	14.5	1.3	3.6
Drty-3.6	1.3	1.7	1.1	30.9	8.3		
GmqP.6	1.8		0.6	5	17.9		4.1
JmqR.6	2		1.1	7.5	17.4	4.7	4.9
Zzq.6	2.9	0.5	0.6	11.2	20.2		3.3
SrbzD.6	0.7	0.3	1.4	10.7	14.3	13	
GxsB.6							
Jz.6	3.6	0.6	1.5	7.8	26.1	0	2.4

**Table 3:** Dissimilarity tests between different microbial groups as defined either temporally or geochemically. P-values indicating significance differences between the groups are highlighted in bold

	MRPP		ANODIS		ANOSIM	
Data sets	δ	P-value	R-Square	P-value	R	P-value
Jan vs. Jun vs. Aug^a^	0.9019	0.742	0.02173	0.369	−0.02149	0.732
Rehai vs. Ruidian	0.8748	**0.004**	0.06081	**0.003**	0.1483	**0.025**
Acid vs. neutral vs. Alkaline	0.8216	**0.001**	0.16176	**0.001**	0.836	**0.001**
Water vs. Sediment^b^	0.8709	**0.001**	0.06437	**0.002**	0.0917	**0.02**

^a^only contained samples that are paired among three sample times (13 samples for each sampling time point).

^b^only contained springs that have paired water and sediment samples at a given time (16 samples (8 for water and 8 for sediment) for January, 14 samples (7 for water and 7 for sediment) for June, and 12 samples (6 for water and 6 for sediment) for August.

**Table 4:** SIMPER analysis identifies the top individual taxa that contributed at least ~1% to the dissimilarity between the microbial communities from three sampling times.

Water: January vs. June vs. August						
Overall average dissimilarity 50.21%						
Genus	Order^a^	Phylum^b^	Contri. %	Mean abund. Jan (%)	Mean abund.Jun (%)	Mean abund. Aug (%)
Hydrogenobacter	Aquificaceae	Aquificae	35.19	70.1	53.9	61.4
Sulfolobus	Sulfolobales	Crenarchaeota	22.54	18.5	16.3	4.19
Persephonella	Hydrogenothermaceae	Aquificae	11.27	0.07	7.51	11.1
		Minor group^c^	4.97	1.48	2.04	7.57
Fervidobacterium	Thermotogales	Thermotogae	4.89	0.49	6.58	0.42
Pyrobaculum	Thermoproteales	Crenarchaeota	4.24	1.02	3.35	3.31
Thermus	Thermales	Deinococcus-Thermus	4.21	1.79	3.99	2.89
Sulfophobococcus	Desulfurococcales	Crenarchaeota	2.67	2.17	2.17	0.92
Caldimicrobium	Thermodesulfobacteriales	Thermodesulfobacteria	1.71	2.45	0.34	0.4
unclassified genus	Thermotogales	Thermotogae	1.51	0.02	2.11	0.12
Desulfurococcus	Desulfurococcales	Crenarchaeota	1.46	1.07	0.22	1.26
unclassified genus		GAL35	1.46	0.04	0.68	1.56
unclassified genus		Crenarchaeota	0.98	0.01	0	1.42
Tepidimonas	Burkholderiales	Beta-proteobacteria	0.96	0	0.1	1.34

Sediment: January vs. June vs. August						
Overall average dissimilarity 83.54%						
Genus	Order^a^	Phylum^b^	Contri. %	Mean abund. Jan (%)	Mean abund. Jun (%)	Mean abund. Aug (%)
Sulfolobus	Sulfolobales	Crenarchaeota	15.93	12.7	24.7	10.4
Hydrogenobacter	Aquificaceae	Aquificae	15.25	29.8	14.1	5.4
Hydrogenobaculum	Aquificaceae	Aquificae	6.08	11.6	4.2	1.67
		Minor group^c^	5.84	5.75	8.77	12.8
Acidicaldus	Rhodospirillales	Alpha-proteobacteria	5.14	6.14	3.69	4.99
unclassified genus		GAL35	4.95	4.3	5.91	3.91
unclassified genus		OP1	4.83	2.98	3.53	8.38
Ignisphaera	Desulfurococcales	Crenarchaeota	4.54	0.18	0.81	10.6
Sulfophobococcus	Desulfurococcales	Crenarchaeota	4.12	2.23	6.08	3.77
Thermus	Thermales	Deinococcus-Thermus	3.5	1.13	1.12	7.21
Desulfurella	Desulfurellales	Delta-proteobacteria	3.46	2.98	2.86	3.83
Persephonella	Hydrogenothermaceae	Aquificae	3.33	0.66	0.51	7.3
Fervidobacterium	Thermotogales	Thermotogae	3.12	0.77	6.16	1.19
Desulfurococcus	Desulfurococcales	Crenarchaeota	2.95	0.09	0.99	6.46
unclassified genus		Armatimonadetes	2.34	3.39	2.23	0.87
unclassified genus		Crenarchaeota	1.72	1.38	2.18	1.22
unclassified genus	Thermoplasmatales	Euryarchaeota	1.65	1.69	2.54	0.19
unclassified genus		Aquificae	1.37	1.62	0.98	1.49
Caldimicrobium	Thermodesulfobacteriales	Thermodesulfobacteria	1.23	2.81	0.47	0.27
Geothermobacterium	Thermodesulfobacteriales	Thermodesulfobacteria	1.19	1.5	0.53	1.41
CandidatusNitrosocaldus	Nitrosocaldales	Thaumarchaeota	1.05	0.95	1.75	0.06
unclassified genus		O1aA90	0.99	1.44	1.14	0.1

aOrder level for most bacteria and archaea and family level for Aquificales.

bPhylum level for most bacteria and class level for archaea and Proteobacteria.

c“minor group” represents the sum of microbial lineages with abundance <5% of the total sequences in each sample.
