# Supplementary material for: Greater temporal changes of sediment microbial community than its waterborne counterpart in Tengchong hot springs, Yunnan Province, China
Source: Sci Rep. 2014 Dec 19;4:7479. doi: 10.1038/srep07479 (PMC5378992; doi:10.1038/srep07479)
Supplement: Supplementary Information — Supplementary tables and figures [file srep07479-s1.pdf]

Greater temporal changes of sediment microbial community than its waterborne counterpart in Tengchong hot springs, Yunnan Province, China

Shang Wang, Hailiang Dong*, Weiguo Hou, Hongchen Jiang, Qiuyuan Huang ,
Brandon R. Briggs, and Liuqin Huang

Table S1 Geochemical parameters determined by Hach kit in the field and major ions determined in lab

Spring ID [a]	NH_4^+-N	NO_2^-+NO_3^-	Fe^{2+}	Total Hydrogen Sulfide	Oxygen	Mg	Fe	Ca	K	Na	Cl	SO_4^{2-}	F
	(μM)				(μg/L)	(mg/L)							
Drty-1.1	71.4	92.9	60.6	3.2	NA	6.7	16.4	48.7	92.7	17.5	6	991.2	0.3
Drty-2.1	7.9	42.9	59.1	0.3	NA	1.2	26.8	12.8	39.8	11.2	bdl [a]	809.9	bdl
Drty-3.1	0	128.6	291.2	22.1	NA	0.2	4.6	1.5	32.8	5.3	8.2	243.2	bdl
GmqP.1	0	53.6	0.2	154.4	NA	0	bdl	bdl	126.8	719.9	658.3	23.6	13
JmqR.1	0	50	0.7	125	NA	0.1	0.5	0.4	134.6	752.8	710.3	19.8	14.1
Zzq.1	0	0	11.5	132.4	NA	0.3	0.2	2.2	34.2	46.9	36.1	103.9	0
SrbzD.1	10.7	67.9	0.7	22.1	NA	0.1	0.1	2	78.2	475.9	374.2	21.3	5.4
GxsB.1	7.1	25	0.2	0.6	NA	4.2	bdl	6.9	46.1	430	166.3	23.6	4.8
Jz.1	0	21.4	0	0.9	NA	4	bdl	8.2	42.6	390.2	146.9	22.2	5.1
Drty-1.6	1309.5±41.2	1.1±0.1	203.1±10.3	43.2±1.7	506±28	4.9	9.7	82	176.9	13.3	19.3	713.4	1.6
Drty-2.6	143±0.1	3.2±0.2	412.2±17.8	61.8±0	624±23	4.1	21.5	63.8	62.2	28.7	8.8	1380.5	1.5
Drty-3.6	71±0.1	1.8±0.1	370.4±41.2	9.1±0.3	650±71	0.6	23.8	22.8	89.2	34.7	4.6	594.2	1.4
GmqP.6	6.7±1.1	84.6±0.1	0	156.8±22.5	246±26	0	0	23.3	215.3	1461	506.5	18.8	12.7
JmqR.6	4.5±1.1	1.4±0.1	0	171.5±8.5	196±74	0	0	22.9	203.6	1386.3	543.2	22.4	13.7
Zzq.6	500±0.2	0.4±0.1	3.5±0.1	1.2±0.2	783±35	0.5	0.5	25.7	37.6	103.1	38.2	91.8	1.4
SrbzD.6	4.8±0.8	12.9±0.1	34.5±2.7	12.1±1.2	487±83	0	0	24.3	126.8	880.7	323	29.1	8
GxsB.6	4.3±0.1	5±0.1	0	0.3±0.1	550±212	4.3	0	36.6	120.5	832	146.2	21.8	6.6
Jz.6	10±0.1	0.7±0.1	0	0.3±0.1	567±60	4.2	0	45.4	115.5	774	137.7	23	6.3
Drty-1.8	1285.7	0.7	175	6.8	1010	17.3	1.7	123	368	45.5	89.4	494.3	6.1
Drty-2.8	214.3	2.9	155	76.5	1100	0.7	9.1	13.6	14.8	14.6	5.8	398.5	0.6
Drty-3.8	142.9	0.4	242.9	11.8	1030	0.7	15.5	5.1	25.7	25.5	4.5	336.2	0.9
GmqP.8	9.3	0.7	0.2	9.4	410	0.4	0	7.7	89.6	790.5	410.9	30.2	12.3
JmqR.8	10	1.1	1.1	10.9	140	0	0	4	100.1	912.8	458.4	19.8	14.4
Zzq.8	250	0.4	0	11.8	1870	0.4	0.6	7	19.5	82.9	36.7	72.1	1.2
SrbzD.8	0.7	NA	0	NA	1563	0.4	1	7.3	63.3	566.6	266.3	39.6	7.8
GxsB.8	5	0.7	0.2	0	1430	3.9	0	23.2	41.7	609.3	111.6	19.6	6.8
Jz.8	5	0.7	0.4	0.9	290	3.8	0	31.8	38.8	561.3	98.2	17	5.7

[a] bdl: below detection limit

Table S2 Microbial diversity indices in January, June, and August sediment and water samples

	Chao1	SD [a]	Shannon	SD	Equitability	SD	Phylogenetic diversity [b]	SD
Drty-1.S.1	48	18	1.48	0.07	0.3	0.01	3.79	0.53
Drty-1.S.6	100	11	3.35	0.06	0.53	0.01	6.28	0.3
Drty-1.S.8	36	10	1.35	0.06	0.29	0.01	2.52	0.3
Drty-1.W.1	42	10	1.8	0.06	0.37	0.01	3.68	0.41
Drty-2.S.1	74	16	3.09	0.07	0.54	0.01	5.42	0.4
Drty-2.S.6	85	24	1.74	0.07	0.31	0.01	4.86	0.65
Drty-2.W.1	63	14	2.79	0.07	0.51	0.01	5.11	0.52
Drty-3.S.1	73	11	3.81	0.06	0.65	0.01	5.74	0.46
Drty-3.S.6	57	16	3.13	0.05	0.59	0.01	4.16	0.6
Drty-3.S.8	75	10	3.09	0.03	0.54	0.01	6.22	0.3
Drty-3.W.6	63	13	2.59	0.07	0.47	0.01	5.57	0.46
Zzq.S.6	77	15	2.38	0.07	0.42	0.01	4.93	0.53
Zzq.S.8	99	16	3.33	0.06	0.55	0.01	7.1	0.41
Zzq.W.1	58	24	2.33	0.07	0.47	0.01	3.02	0.52
Zzq.W.6	46	19	1.08	0.07	0.23	0.01	3.16	0.59
Zzq.W.8	86	18	2.68	0.09	0.46	0.01	5.22	0.64
GmqP.S.1	89	23	2.16	0.09	0.38	0.01	4.78	0.77
GmqP.S.6	160	34	2.94	0.09	0.46	0.01	7.35	0.68
GmqP.W.1	152	30	2.99	0.1	0.47	0.01	7.45	0.71
GmqP.W.6	128	45	1.86	0.08	0.33	0.01	4.72	0.83
GmqP.W.8	369	48	3.9	0.12	0.52	0.01	12.21	0.95
JmqR.S.1	110	26	2.67	0.09	0.45	0.01	5.95	0.5
JmqR.S.6	124	24	3.32	0.08	0.53	0.01	6.6	0.6
JmqR.W.1	158	32	3.41	0.09	0.53	0.01	7.45	0.71
JmqR.W.6	121	40	2.31	0.09	0.39	0.01	5.35	0.76
JmqR.W.8	124	46	2.09	0.08	0.37	0.01	5.29	0.79
SrbzD.S.1	212	31	4.41	0.09	0.63	0.01	9.98	0.66
SrbzD.S.6	223	27	4.86	0.08	0.67	0.01	10.69	0.59
SrbzD.S.8	259	26	6.39	0.06	0.85	0.01	12.15	0.6
SrbzD.W.6	221	43	3.6	0.1	0.53	0.01	8.41	0.73
SrbzD.W.8	231	34	4.02	0.1	0.57	0.01	9.45	0.78
GxsB.S.1	211	26	5.37	0.08	0.75	0.01	9.95	0.62
GxsB.S.8	135	17	3.45	0.06	0.53	0.01	9.04	0.43
GxsB.W.1	60	16	2.87	0.07	0.53	0.01	3.77	0.52
GxsB.W.6	93	34	2.11	0.08	0.38	0.01	4.53	0.7
GxsB.W.8	83	26	1.82	0.08	0.33	0.01	4.57	0.6
Jz.S.8	154	17	4.92	0.05	0.72	0.01	7.75	0.41
Jz.W.1	116	27	2.34	0.09	0.39	0.01	5.46	0.51
Jz.W.6	106	28	2.39	0.08	0.4	0.01	5.05	0.59
Jz.W.8	154	23	3.11	0.09	0.47	0.01	7.93	0.61

[a] SD: standard deviation

[b] The simplicity of PD (phylogenetic diversity) means that it readily can be calculated directly from the cladogram, for a given subset of taxa, by adding together the appropriate branch lengths.

Figure. S1

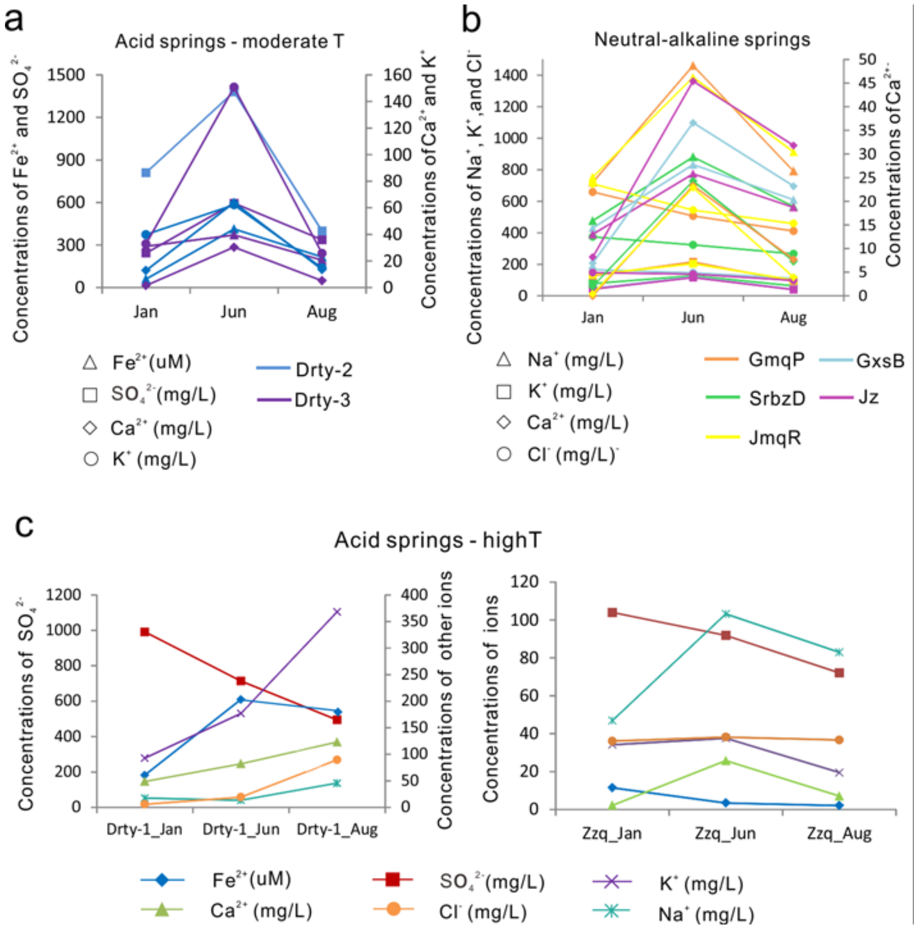

Figure. S1 Temporal variations in ion concentrations of waters in some representative springs. **a.** Major ion concentrations in the moderate-temperature acidic springs. **b.** Major ion concentrations in the neutral-alkaline springs. **c.** Non-uniform temporal variations in major ion concentrations of the high-temperature acidic springs.

Figure. S2

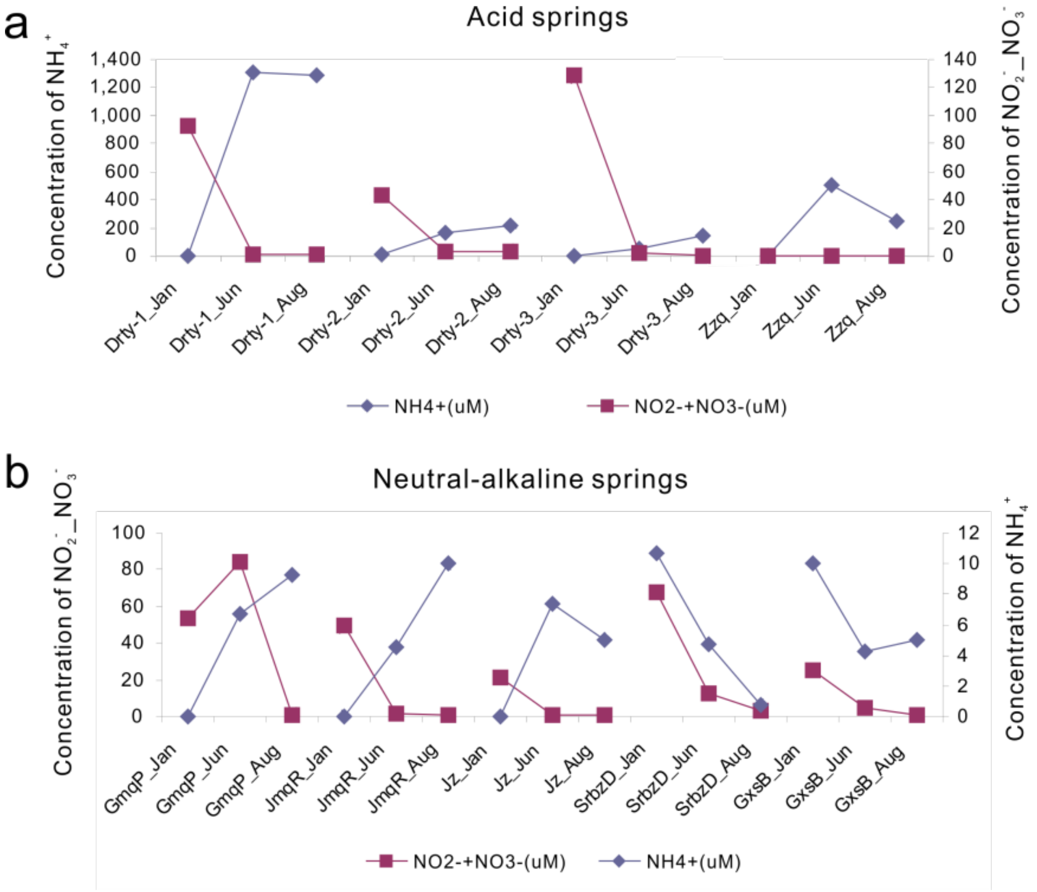

Figure. S2 Temporal variation patterns observed for NH_4 and NO_2+NO_3 concentrations. a. NH_4 and NO_2+NO_3 concentrations determined in each acidic spring at three sampling time points. b. N-related parameters NH_4 and NO_2+NO_3 concentrations in neutral-alkaline spring waters at the three sampling time points.

Figure. S3

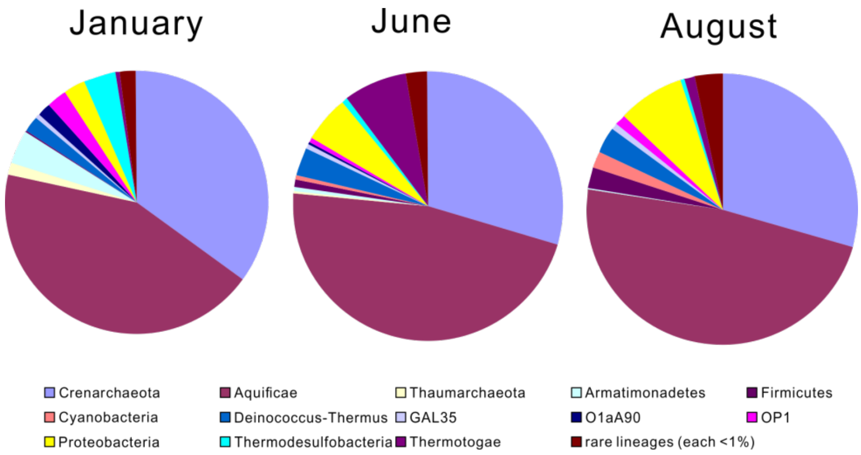

Figure. S3 Overall community composition at each sampling time point.

Figure. S4

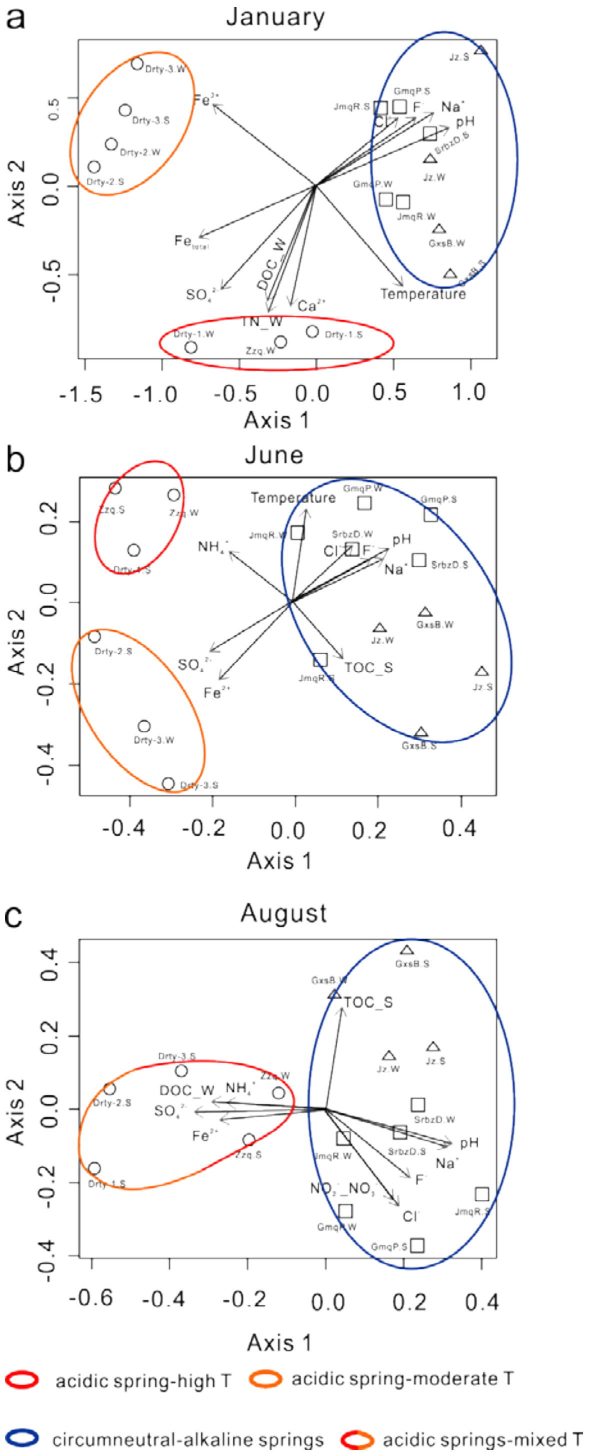

Figure. S4 NMDS ordinations based on Bray-Curtis dissimilarity for January (a), June (b) and August (c) communities. Different symbols represent samples from distinct pH range, with triangles for neutral springs, circles for acidic springs, and squares for alkaline springs, respectively. The samples that cluster together indicate similar microbial community structure. The overlaid environmental factors are those that are significantly correlated with microbial community structure. The length of the vector is proportional to the correlation strength.
